# Effects of Diet on Sleep: A Narrative Review

**DOI:** 10.3390/nu12040936

**Published:** 2020-03-27

**Authors:** Hannah Binks, Grace E. Vincent, Charlotte Gupta, Christopher Irwin, Saman Khalesi

**Affiliations:** 1 Central Queensland University, School of Health, Medical and Applied Sciences, Melbourne 3000, Victoria, Australia; hannah.binks@cqumail.com; 2 Central Queensland University, Appleton Institute, Adelaide 5034, South Australia, Australia; g.vincent@cqu.edu.au (G.E.V.); c.gupta@cqu.edu.au (C.G.); 3 Griffith University, School of Allied Health Sciences, Menzies Health Institute Queensland, Gold Coast 4222, Queensland, Australia; c.irwin@griffith.edu.au; 4 Central Queensland University, School of Health, Medical and Applied Sciences and Appleton Institute, Brisbane 4000, Queensland, Australia

**Keywords:** sleep quality, sleep duration, nutrients, dietary supplements, food, adults

## Abstract

Many processes are involved in sleep regulation, including the ingestion of nutrients, suggesting a link between diet and sleep. Aside from studies investigating the effects of tryptophan, previous research on sleep and diet has primarily focused on the effects of sleep deprivation or sleep restriction on diet. Furthermore, previous reviews have included subjects with clinically diagnosed sleep-related disorders. The current narrative review aimed to clarify findings on sleep-promoting foods and outline the effects of diet on sleep in otherwise healthy adults. A search was undertaken in August 2019 from the Cochrane, MEDLINE (PubMed), and CINAHL databases using the population, intervention, control, outcome (PICO) method. Eligible studies were classified based on emerging themes and reviewed using narrative synthesis. Four themes emerged: tryptophan consumption and tryptophan depletion, dietary supplements, food items, and macronutrients. High carbohydrate diets, and foods containing tryptophan, melatonin, and phytonutrients (e.g., cherries), were linked to improved sleep outcomes. The authors posit that these effects may be due in part to dietary influences on serotonin and melatonin activity.

## 1. Introduction

Sleep is essential for maintaining immune health [1], restoring energy [2], and memory consolidation [3]. Achieving good sleep quality (i.e. a low sleep latency, a low number of awakenings >5 min, low waking after sleep onset, and good sleep efficiency) [4] and duration (i.e., 7–9 h/day) [5,6] is necessary for overall health and wellbeing. Despite the importance of obtaining adequate sleep, approximately 45% of Australian and American adults do not achieve the recommended 7–9 h of sleep per night [7,8]. Thus, identifying simple lifestyle strategies that assist with improving sleep outcomes in the general population is paramount.

Prior to exploring factors that may influence sleep, it is important to understand how sleep outcomes are typically assessed under research conditions. The gold standard approach is polysomnography (PSG), which is used to detect physiological activity during sleep, such as brain waves, eye movements, and muscle and heart activity. Inadequate sleep may influence sleep micro-architecture, leading to changes in any of the four sleep stages: Non- rapied eye movement (NREM) sleep stages N1, N2, and N3, and rapid eye movement (REM) sleep. These changes may include some stages occurring sooner, an increase in the intensity of a stage, or a decrease in the amount of time spent in a stage [9]. For example, shortened sleep has been linked to reduced REM sleep, which can then lead to daytime cognitive dysfunction [9,10]. Actigraphs are wrist-worn monitors that are also often employed to objectively assess sleep, particularly in field studies due to their low cost and feasibility in longitudinal research [11,12]. Actigraphy has been validated as a useful measure for sleep, despite potentially over-estimating total sleep duration [12]. Sleep metrics can also be obtained subjectively through questionnaires and other self-reporting methods (e.g., sleep diaries). For example, the Pittsburgh Sleep Quality Index (PSQI) is a validated subjective sleep quality questionnaire that is frequently used [13]. Irrespective of the technique employed, the premise of these assessments is to diagnose and characterize sleep issues and determine the effectiveness of treatment interventions. Since inadequate sleep (duration and/or quality) can negatively affect several physiological and psychological processes, the measurement of sleep is therefore important to understand factors that may influence sleep outcomes.

A factor that may have an impact on sleep is dietary intake, as both play an important role in maintaining long-term health and wellbeing [14]. The relationship between dietary intake and sleep has gained considerable scientific attention in recent years, largely in an effort to understand how specific dietary factors directly influence sleep outcomes. For example, improvements in sleep parameters (e.g., increased sleep time and efficiency, and decreased sleep latency) have been identified following the consumption of tryptophan [15]: an amino acid present in foods such as milk [16]. Conversely, the depletion of tryptophan has been shown to reduce sleep quality [17]. The mechanism for this centers around tryptophan competing with other large neutral amino acids (e.g., valine, leucine, isoleucine, tyrosine, and phenylalanine) to cross the blood brain barrier, where it is converted to serotonin, the precursor to the sleep-promoting hormone, melatonin [18]. Other research has also demonstrated that consuming foods containing high concentrations of melatonin and serotonin (e.g., cherries [19]) is associated with improvements in sleep duration and quality [20]. With this in mind, developing a better understanding of the dietary factors that directly impact sleep outcomes is important. 

While several reviews examining the impact of diet on sleep are available, their conclusions have largely been drawn based on studies of clinical populations [21,22,23]. Severe sleep-related disorders (e.g., insomnia) are associated with comorbidities such as depression [24] and stress [25], and thus may confound the relationship between diet and sleep. However, even participants without clinically diagnosed sleep disorders may experience poor sleep due to factors such as work and family commitments, and the late-night use of technology (i.e., smartphones, tablets, and television) [26]. Therefore, the aim of this narrative review is to clarify the effects of diet (specifically the consumption of foods containing ingredients (e.g., tryptophan) that may promote sleep) on sleep metrics (i.e., sleep duration and sleep quality) in otherwise healthy adults. This is important step in determining diet-related guidelines that may be most beneficial for improving the sleep of healthy adults.

## 2. Materials and Methods 

### 2.1. Literature Search

This narrative review searched for sleep and diet research conducted in otherwise healthy adults (i.e., irrespective of BMI classification). Although narrative, the source articles were identified using a systematic search strategy. The online databases Cochrane, MEDLINE (PubMed), and Cumulative Index of Nursing and Allied Health Literature (CINAHL) were searched from 1990 to August 2019 for eligible studies. Key search terms, including MeSH terms, were determined in accordance with the PICO method: “adults” for participants; “diet”, “food”, “nutrients”, and “dietary supplements” for inputs; and “sleep”, “sleep quality”, and “sleep duration” for outcomes.

### 2.2. Study Eligibility and Sselection

Studies were considered eligible if they: 1) were interventional studies, and 2) included adults (≥18 years of age) as subjects. Studies were excluded if they: 1) used alcohol or other sleep-inducing substances as part of an intervention, 2) conducted their study in populations with acute or chronic diseases (including clinically diagnosed sleep-related conditions such as insomnia), or 3) did not have full text articles available in the English language. Studies were also excluded if they investigated the effect of sleep loss (i.e., restriction/deprivation) on dietary intake, or combined dietary intake with additional interventions such as exercise, and there were no arms to control for the effect of the combined therapy.

The preliminary screening of studies was completed by reviewing the titles and abstracts to assess eligibility. Studies that did not align with the inclusion and exclusion criteria at this point were excluded. Of the remaining studies, the full text articles were reviewed to determine inclusion. Finally, a manual review of the reference lists of included studies was undertaken to ensure all appropriate studies were sourced.

### 2.3. Data Extraction and Synthesis

Methodology characteristics, outcomes and effects were extracted from the eligible studies. To classify eligible studies based on emerging themes and similarities between interventions, the extracted data included subject characteristics (such as body weight status: normal, overweight, or obese), length of follow up, dietary source (food items, whole diets, or dietary supplements), dose, sleep outcomes (sleep quality, sleep duration, and sleep architecture), and the methods used to measure outcomes (polysomnography [PSG]/electroencephalography [EEG], actigraphy, and subjective measures). A narrative synthesis reviewed the effects in the identified themes.

## 3. Results

**Figure 1:**
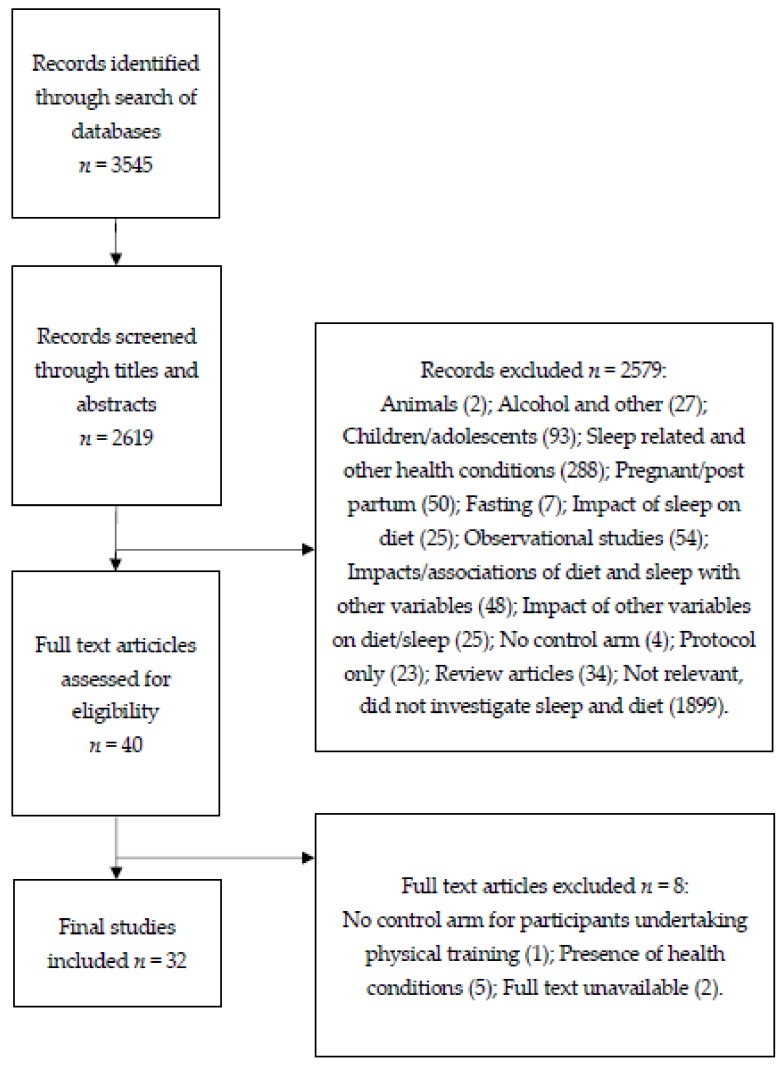
A diagram showing the selection process for articles.

Following the literature search, 3545 articles were identified. After excluding 3512 articles (926 duplicates, 1899 that did not investigate both diet and sleep, two animal studies, 27 that used alcohol or other sleep-inducing substances, 93 that used children/adolescents as participants, 293 that reported sleep-related and other health conditions, 50 that used pregnant or post-partum participants, seven that investigated effects of fasting, 25 that investigated the impact of sleep on diet, 54 that were observational studies, 48 that investigated the effects or associations of diet and sleep on or with other variables, 25 that investigated the effects of other variables on diet and sleep, five that had no control arm for combined interventions, 23 that were protocols only, 34 that were review articles, and two articles for which the full text was not available), 32 articles that met the inclusion criteria remained and were included in the final review. Figure 1 further outlines the selection process used. A narrative analysis of the studies revealed four key themes: tryptophan consumption/depletion, dietary supplements, food items, and macronutrients. The results are classified according to these four themes. 

### 3.1. A Summary of the Included Studies

#### 3.1.1. Tryptophan Consumption and Depletion

Five studies were classified under this category based on the investigation of the effects of either tryptophan consumption [15,27] or acute tryptophan depletion [17,28,29] on sleep in otherwise healthy adults. All of the studies examining tryptophan depletion used PSG to measure sleep, while the studies investigating tryptophan consumption utilized actigraphy [15] and sleep diaries [27]. Table 1 summarizes the characteristics and outcomes of these studies.

Tryptophan-containing foods improved sleep indices compared with controls [15,27]. For example, compared with controls, an increase in total sleep time, sleep efficiency, and immobile time was noted after a week of tryptophan-rich (60 mg) cereal consumption in older adults [15]. Furthermore, participants also experienced less difficulty in falling asleep and less waking at night, total activity, and fragmented sleep. Similar improvements in sleep were noted in another study of middle-aged adults after 19 days of tryptophan-rich (70 mg) egg-white protein hydrolysate formulation consumption [27].

The mechanisms underlying the aforementioned observations relate to the relationship between tryptophan depletion and serotonergic function. Reducing the availability of tryptophan prevents the synthesis of serotonin and subsequently reduces sleep quality [30]. The results of three studies investigating tryptophan depletion on sleep quality elicited mostly similar effects on sleep parameters. It has been reported that significant decreases in total sleep time, sleep efficiency, and time spent in stage N2 occur after a 100% tryptophan-free drink compared with a 25% tryptophan-free drink [17]. Consuming the 100% tryptophan-free drink led to longer sleep onset latency, and both drinks reduced REM latency. Increased wake periods and wake percentages for tryptophan depletion was found compared to baseline and placebo, but there was not a reduction in overall sleep efficiency compared with baseline [28]. Similarly, however, they reported significantly less time in stage N2 sleep, and more time in stage N1 sleep than with the placebo. Furthermore, tryptophan depletion at mid-morning, as opposed to in the evening, leads to a significant increase in the arousal index, REM sleep onset latency, and REM density following the consumption of the 100% Tryptophan free drink, but no other significant effects on sleep were observed [29].

#### 3.1.2. Dietary Supplements

Thirteen studies investigated the effect of consuming dietary supplements on sleep (Table 2). The methods employed to measure sleep outcomes varied across studies. Seven studies used subjective measures (i.e., diaries/logbooks and sleep questionnaires) [31,32,33,34,35,36,37], while one study used PSG/EEG [38,39], and five studies used a combination of PSG/EEG or actigraphy, and subjective measures [39,40,41,42,43].

Overall, studies were highly heterogenous with respect to the dietary supplement(s) administered and the duration of the interventions. Nonetheless, the data suggest that some dietary supplements may be efficacious for improving sleep quality (outlined in the sub-sections below). 

##### Zinc

One study examining zinc supplementation in individuals with below-optimal zinc levels (≤ 79.9 μg/dL) observed improvements in global sleep quality scores on the PSQI, subjective sleep quality, and sleep onset latency scores [32]. A separate study, which administered astaxanthin (a keto-carotenoid) in combination with zinc [43], observed a significant improvement in sleep onset latency, but no effects on total sleep time or sleep efficiency. 

##### B Vitamins

Three studies examined the effect of consuming B vitamins (in isolation or in a multivitamin supplement) on sleep. The consumption of a vitamin B complex resulted in lower subjective reports of sleep quality and higher tiredness upon waking compared with consumption of a vitamin B6 supplement or placebo [31]. Likewise, it was found that the consumption of a liquid nutrient supplement drink (comprising a wide range of nutrients including B vitamins and antioxidants) resulted in better sleep compared with a placebo [35]. Conversely, Sarris, et al. [34] observed non-significant reports of improved sleep with the consumption of a multivitamin supplement containing B vitamins. 

##### Polyphenols

Polyphenols are a group of plant compounds including flavonoids, phenolic acids, stilbenes, and lignans. The effect of a blend of polyphenol compounds (known as HolisFiit and consisting of flavonoids, as well as natural caffeine and vitamin B3) on sleep quality was studied by Romain, et al. [33]. Compared to the placebo group, those ingesting HolisFiit reported lower sleep disturbances, and greater total sleep duration and sleep quality at the end of 16 weeks of supplementation. However, the ingestion of pure resveratrol (a stilbenoid) elicited no effect on sleep in young adults over a 28-day period [36]. On the other hand, it was found that a group of poor sleepers who were administered phlorotannin (a supplement derived from brown seaweed) experienced decreased waking after sleep onset and total wake time compared with those administered the placebo [41]. 

##### Crocetin

Two studies examined crocetin (a carotenoid compound) supplementation on sleep, using actigraphy [40] and PSG [42], combined with subjective measures. Significantly fewer waking episodes, as recorded by the actigraphy data, occurred when participants consumed crocetin compared with the placebo [40]. On the other hand, participants reported feeling refreshed, and had less sleepiness upon waking, following crocetin administration, compared with placebo administration, with no differences in waking found in the PSG recordings [42]. However, taking crocetin was associated with a higher delta power (which is negatively linked with wake episodes during REM latency) [42].

##### Chlorogenic Acids

One study examined the effect of chlorogenic acids (CGA) derived from green coffee beans (caffeine extracted) on sleep [38]. Participants who consumed CGA experienced shortened sleep onset latency and higher delta power in the first hour of sleep compared with controls, demonstrating a potential benefit of CGA for sleep quality.

##### *Chlorophytum bovivilianum* (Root) and Velvet Bean

One study examining the effect of consuming a *Chlorophytum bovivilianum* and velvet bean supplement observed reduced sleep onset latency as well as improved global sleep quality scores on the PSQI, subjective sleep quality, sleep duration, habitual sleep efficiency, and sleep disturbance scores, compared to those that did not consume the supplement [37].

##### γ-Aminobutyric Acid and *Apocynum venetum* Leaf Extract

The amino acid, γ-aminobutyric acid (GABA), and *Apocynum venetum* (herb) leaf extract (AVLE) were examined both separately and in combination compared to placebo, to identify changes in sleep indices [39]. GABA significantly reduced sleep onset latency, while AVLE increased non-REM sleep time, although minimal effects of AVLE were observed on delta waves. The combination of these ingredients did not provide a synergistic effect.

#### 3.1.3. Food Items

Nutrients are likely to benefit general health more when consumed as part of food rather than as supplements [44]. Despite this, the effect of consuming specific foods on sleep outcomes in healthy individuals has received limited attention. Table 3 provides a summary of the four studies investigating the consumption of specific food items on sleep. All of the studies used actigraphy to measure sleep metrics, three of which combined this with subjective measures.

One study investigated the sleep-promoting effects of consuming different cultivars of Jerte Valley cherries (sourced from Spain) in middle-aged and elderly individuals [45]. The effect of each cultivar on sleep quality varied, likely due to the differing levels of serotonin and melatonin [19]. However, in general, significant improvements were found in middle-aged participants for total sleep time, sleep efficiency, the number of awakenings, total nocturnal activity, assumed sleep, and sleep onset latency. Meanwhile, older adults experienced decreases in the number of awakenings, immobility, and sleep onset latency, and increases in assumed sleep. In a later study, the impact of consuming a Jerte Valley product containing four combined cultivars of cherries was explored in young, middle-aged, and elderly participants compared with placebo controls. Improvements across several sleep indices (i.e., increased actual sleep time and immobility, fewer awakenings and decreases in sleep onset latency) were observed, with greater improvements identified in the middle-aged and elderly groups [20]. Likewise, another separate study investigating the consumption of Montmorency tart cherry juice reported that participants spent less time napping and more time sleeping, and had higher total sleep efficiency scores compared with baseline and placebo [46].

One study has also examined the effect of consuming seafood on sleep outcomes, whereconsuming zinc-rich oysters and astaxanthin-containing krill resulted in improved global sleep quality scores from the PSQI and sleep onset latency [43]. 

#### 3.1.4. Macronutrients

Ten studies investigated the effects of manipulating the macronutrient content of dietary intake on sleep outcomes (Table 4). Across the studies, the intervention was either a high or low carbohydrate (CHO); high protein; or combination of high CHO, high protein, and high fat diet/meal; while the methods employed to assess sleep outcomes were heterogenous (i.e., *n* = 5 used PSG, *n* = 2 used Actigraphy, and *n* = 5 used subjective ratings).

A number of the studies summarized in Table 4 manipulated CHO intake as part of the intervention. In one study, healthy men experienced a shortened sleep onset latency when they were provided with a high glycemic index (GI) meal that was consumed four hours before bedtime, compared with the consumption of a low GI meal, or a high GI meal one hour before bedtime [48]. Meanwhile, in a separate study, reduced sleep onset latency was observed following the consumption of a high CHO diet over a four-day period [50]. In contrast, however, one study did not observe significant changes in sleep onset when a high CHO meal was consumed four hours prior to bedtime, compared with a high fat intake, but reported significantly reduced time in N3 (the deep, restorative portion of sleep) during the first sleep cycle [51]. However, the total time in N3 across all sleep cycles was not different between the two diets.

Two studies explored the effects of ketosis (a physiological state induced by a diet typically characterised by a low CHO and high fat intake) on sleep outcomes. One study found that sleep parameters did not differ between the acute and ketosis phases of a very low CHO, high fat, and high protein diet [49]. However, increases in the percentage of N3 and decreases in the percentage of REM sleep were observed following ketosis compared with control. In a separate study involving the provision of a very low calorie, ketogenic diet to obese participants for four months, an improvement in subjective sleepiness was observed during the reduced (but not maximum) ketosis phase; however, no changes in other self-reported sleep quality measures were identified [55].

Several studies summarized in Table 4 examined the influence of manipulating other macronutrients (i.e., fat and protein) as well as consumption patterns (i.e., ad libitum vs. controlled intakes) on sleep. It was demonstrated that ad libitum eating over 3 days in men and women led to a reduction in N3 and increased the time taken to fall asleep, indicating poorer sleep than with controlled eating [53]. Saturated fat intake was associated with less time spent in N3, while sugar and other CHOs (excluding fibre) were linked with increased arousal. Consuming fibre was associated with less time spent in N1, the lightest stage of sleep, and more time in N3. In another study, participants experienced fewer wake episodes following high protein meals; however, no significant findings were reported between sleep outcomes and high fat intake [50]. Conversely, in a similar study, a high fat diet consumed over four days was associated with better self-reported sleep than a high CHO diet or high fat diets [52]. Furthermore, the actigraphy data in this study showed that the consumption of high CHO meals was associated with shorter waking times.

Two studies summarized in Table 4 explored the impact of energy-restricted, protein-supported dietary intakes on sleep outcomes. Improvements in participants’ sleep were found following four weeks of an energy-restricted diet with 20% protein intake, compared with 10% and 30% protein intakes [54]. However, the source of protein did not affect sleep parameters (beef/pork vs. soy/legumes). The same authors also indicated anecdotal reports of improved global sleep quality scores, as measured by the PSQI, at week 12 and week 16 in a separate study intervention involving a high protein diet vs. a normal protein diet [54]. This was thought to be the result of increased tryptophan and tyrosine. However, the high energy content of a single evening meal (fat 37%, protein 21%, CHO 42%) consumed 2 to 3 h prior to bedtime had no effect on objective or subjective sleep parameters [47].

## 4. Discussion

This narrative review explored the sleep-promoting effects of food and supplement intake in otherwise healthy adults. Overall, the collective works included in this review suggest that the consumption of certain nutrients (e.g., tryptophan), food items (e.g., cherries), and dietary supplements (e.g., zinc), and manipulating some dietary components (i.e., macronutrient and energy composition) can influence (i.e., disrupt or improve) sleep outcomes. However, the effect appears to vary based on a number of factors (e.g., the type of food/supplement, the magnitude of dietary manipulation, etc.).

A diet poor in tryptophan appears to impair sleep. Acute tryptophan depletion affects the synthesis of serotonin and can reduce REM sleep onset latency in normal sleepers [17]. Inconsistencies noted between the tryptophan depletion studies in the current review are likely to be a result of methodological differences (e.g., the time and duration of tryptophan depletion before bed, familiarization to environments, and participant characteristics). As discussed in this review, the ingestion of tryptophan can improve sleep [15], but this is largely dependent on its ability to cross the blood-brain barrier [28]. This mechanism is often impeded by dominating plasma levels of other large neutral amino acids (LNAAs), which are more abundant than tryptophan [56]. High CHO meals can increase the tryptophan to LNAA ratio [56]. An increase of plasma glucose drives insulin secretion and the consequent removal of circulating LNAAs [56]. Tryptophan is excluded from this process, therefore leaving it in a favourable position to cross the blood-brain barrier [56] and consequently convert into serotonin. This could also partly explain consistent findings for improved sleep following the ingestion of high CHO meals in the current review, though these studies did not directly examine the mechanism. However, it was reported that urinary measures of 6-sulfatoxymelatonin peaked following the consumption of the high GI meal compared with the low GI meal [48]. As a metabolite of melatonin, 6-sulfatoxymelatonin has previously been observed to rise with the supplementation of tryptophan and decrease with tryptophan depletion [57].

Despite the limited number of studies, the effect of consuming specific ‘whole foods’ on sleep has promise. For instance, the consumption of Jerte Valley cherries and Montmorency tart cherries has demonstrated efficacy in improving several objective indices of sleep in young, middle-aged, and elderly individuals [20,45,46]. This is likely attributable to the melatonin, serotonin [19], and other phytonutrient content [58] within the fruit. The consumption of tart cherries has also been anecdotally reported to improve sleep quality in a sample of elderly insomniacs [59]. Collectively, these findings suggest that cherry consumption could improve sleep outcomes. Likewise, consuming certain seafood ingredients (i.e., zinc-rich oysters) may have beneficial effects on sleep. Nonetheless, with relatively few studies—on a limited selection of whole foods—conducted to date, further research is warranted. For example, melatonin and polyphenols such as resveratrol are also found in grape products, mainly in the skin (and to a lesser extent in the seed and whole berry) [60], which may provide an avenue for potential future research directions.

Collectively, a growing body of research supports the beneficial effects of consuming some dietary supplements on sleep outcomes (i.e., sleep onset latency, sleep quality, sleep quantity, and feelings of alertness upon waking). For example, daily phlorotannin supplementation reduced wake after sleep onset and total wake time. Phlorotannins, polyphenolic compounds in marine plants, have previously been demonstrated to promote NREM sleep and reduce sleep onset latency in mice [61]. The potential sleep-promoting benefit of phlorotannins has been identified through their ability to bind to GABA_A_-benzodiazepine receptor sites, which may therefore may assist in obtaining adequate sleep, since GABA is associated with sleep regulation [61]. Decreases in sleep onset latency and increases in NREM were also observed with GABA supplementation, and the administration of a herb leaf extract in the study of Yamatsu, et al. [39]. Two studies examining the effects of zinc supplementation (and the consumption of zinc-enriched foods) observed effects on sleep onset latency, among other indicators. While zinc has a known role in many physiological processes, its involvement in sleep is a little less clear [62]. However, using survey data from the 2007-2008 National Health and Nutrition Examination Survey, Grandner, et al. [63] reported that subjects who slept less than 5 h per night had lower intakes of zinc, potentially suggesting its involvement in sleep regulation. Additionally, sleep onset has been linked to *Chlorophytum bovivilianum* (root) and velvet bean supplement. A combination of these has been previously reported to increase growth hormone release post-consumption [64]. Since growth hormone is naturally released when the body is promoting sleepiness [65,66], *Chlorophytum bovivilianum* (root) and velvet bean may help improve sleep onset latency. 

The current review is a narrative review with a systematic search and a narrative analysis. This allowed for a thorough search of the research databases and discussion of the four themes of research that emerged. This approach overcomes the evidence bias that may be present in a traditional narrative review. Further, this review focused on the evidence of the effect of food on the sleep of healthy adults. Therefore, the inclusion of animal, molecular, or other populations (e.g., patients with clinical conditions) were outside of the scope of the aims of the current study. Given the systematic nature of the search, we are confident that the key nutrition and dietary themes that are currently linked to increased sleep behaviour in healthy adults have been identified. It is also important to consider that the variations in intervention characteristics and methodology observed in the included studies limit the generalization of findings. Similar to other systematic and narrative reviews, included studies in this review were selected based on a priori selection criteria to reduce the heterogeneity between study characteristics. However, this study aimed to find all food and dietary supplements that affect sleep in healthy adults. Given the scope of the study, the inclusion of a wide variety of interventions was expected.

A strength of the literature included in this review is the variety of countries (Western and non-Western) included. This is important as there are cultural differences in meal timing and nutrient intake, which may influence the impact on sleep. Together, these studies demonstrate that, cross-culturally, sleep is influenced by nutrient intake [67,68]. The majority of the studies included in this review utilized validated subjective methods for measuring sleep, with the PSQI the most frequently used. However, self-reported sleep tends to be overestimated [69]. Future studies should consider PSG techniques where possible, as these measures provide more accurate objective measures of sleep duration and sleep quality. In this review, 20 of the 32 studies utilized objective measures, frequently with subjective methods, possibly due to the relative convenience of collecting subjective measures. Intervention characteristics and methodology also varied, which may limit the generalizability of findings. Furthermore, extraneous factors that may influence sleep and dietary intake, including BMI, work schedule, physical and mental health, and light exposure, should be considered in future studies. A limitation of our search criteria, namely the focus on human studies rather than animal models, is the smaller sample sizes of these clinical studies. Future research could include animal studies and larger sample sizes, in addition to investigating the mechanisms and pathways behind the relationship between dietary intake and sleep. Future studies should also consider meal timing and background diet as a potential confounder on the impact of a dietary intervention on sleep

Although this narrative review has highlighted certain foods and nutrients with the potential to promote sleep, when recommendations for nutrient intake are made, it is also important to consider timing of food intake, a concept termed chrononutrition [70]. Chrononutrition combines the health, behavior, and chronobiology disciplines to understand the optimum timing of food intake for health and performance [71]. Eating at a time that contradicts our circadian system, such as nocturnal eating, can entrain rhythms in peripheral clock tissues, such as the liver, and cause misalignment for metabolic processes, glucose homeostasis, gastrointestinal motility, and digestive processes [72,73]. On the other hand, time-restricted feeding (i.e., shortening the feeding window during the day) or regular meal-timing (in particular, breakfast) can entrain circadian clocks, preventing the development of metabolic conditions [73]. In addition, consuming certain nutrients (e.g., caffeine) and melatonin supplements can elicit phase-shifting responses of the sleep/wake cycle, so the timing of intake is important to consider. In the present review, greater improvements of sleep onset latency occurred when high GI meals were consumed four hours prior compared with one hour prior to bedtime [48]. This finding is supported by previous research demonstrating peak circulating tryptophan to LNAA ratios in the 2–4 hour period after the consumption of a high CHO meal [74]. Though the current review did not specifically aim to investigate studies examining these effects, it appears to be an important consideration when unpacking the complex associations between diet and sleep, and their metabolic consequences. Research exploring the effects of dietary intake on sleep should consider the timing of dietary interventions in relation to sleep periods.

The current narrative review evaluated a wide range of studies highlighting an array of new evidence in this field. However, given the number of variables associated with sleep, dietary intake, and dietary behaviour, the study methodologies were largely heterogenous, and we were therefore unable to conduct a quantitative analysis (i.e., meta-analysis) of the collective evidence. Methodological differences were observed mainly for the intervention type (i.e., foods, supplements, or nutrients), length of intervention period, and assessment of sleep outcomes, with most studies employing subjective sleep metrics, which may be subject to a greater level of bias or inaccuracy. More research using objective sleep metrics (i.e., data from validated sleep recording devices) either in laboratory-based controlled environments or naturalistic settings are needed. Furthermore, in many of the studies in this review, it was not possible to conclude regarding the effects of individual nutrients, given the number of nutrients consumed together. Unlike previous reviews, this study only included original investigations employing healthy participants with no clinical diagnosis of sleep disorders, thereby removing the potential effect of comorbidities that may affect sleep, and allowing for an increased generalization of the results.

## 5. Conclusions

This narrative review highlights research linking dietary intake and sleep outcomes. The consumption of high CHO diets, foods containing tryptophan, melatonin, and phytonutrients (i.e., cherries) indicates promising results for improved sleep quality and quantity. Further research is needed to understand the mechanisms underlying many of these effects; however, it is likely due to a dietary influence on serotonin and melatonin activity. Future research should ensure the consistency of dietary measures used and employ both objective (e.g., PSG/EEG) and validated sleep assessment techniques, to enhance the accuracy of the results and allow for better comparisons between studies. 

## Figures and Tables

**Table 1:** The study characteristics and outcomes for the investigations between tryptophan consumption, tryptophan depletion, and sleep.

Author (Year)	Design; Location	Duration; Age; Participants (m/f)	Intervention/Control	Sleep Measurements	Outcomes
Bravo (2013) [15]	SB, P; Spain	3 wk; 55–75 y; 35 (9/26)	30 g tryptophan-enriched cereals (containing 60 mg tryptophan)/control Subjects consumed their habitual diet posttreatment week (third week) with no cereals.	Actigraphy	Increase in actual sleep time, SE and immobile time and decrease in SOL, wake bouts, total activity and fragmentation index for intervention compared with control and habitual diet.
Bhatti (1998) [17]	RC, DB, CO	6 dy; 21–53 y; 11 (11/0)	100% tryptophan-free amino acid drink/control	PSG	Significant reduction in REM latency for both intervention and control compared to baseline. Significant increase in REM sleep time and REM% for control, non-significant increase in REM% for intervention compared to baseline. Significant increase in SOL for intervention compared to baseline. Significant decrease in TST, SE and stage 2 min for intervention compared to control. Significant increase in WASO for control compared to intervention. No significant difference in NREM measures.
Mohajeri (2015) [27]	RC, DB, P; UK	19 dy; 45–65 y; 59 (0/59)	lumiVida™ (egg-white protein hydrolysate formulation containing ~70 mg tryptophan)/placebo	Subjective (sleep diaries)	Non-significant improvement in sleep quality for intervention compared with control. Non-significant increase in difficulty in getting up out of bed during trial for intervention.
Voderholzer (1998) [28]	RC, DB, CO, P; Germany	2 × 4 dy; 23–55 y; 12 (6/6)	Low protein diet + one capsule of 0.5 g L-tryptophan (day 3 and 4), tryptophan-free amino acid drink (day 4)/placebo	PSG	Significant increase in amount of wake periods, wake times and REM density for tryptophan depletion compared with baseline. Significant decrease in stage 2 sleep for tryptophan depletion compared with baseline.
Arnulf (2002) [29]	RC, DB, CO, P; France	3 dy; 18–39 y; 18 (7/11)	100% tryptophan-free amino acid drink + capsules containing methionine, arginine and cysteine/placebo	PSG	Significant increase in arousal index, REM sleep onset latency and REM density for intervention. Non-significant increase in sleep N1 and N2. No significant difference in SOL, TST, total sleep period, duration of WASO, and 3–4 of NREM sleep or REM sleep for intervention.

CO – Crossover; DB – Double blind; P – Placebo; NREM – Non-rapid eye movement; RC – Randomised controlled; REM – Rapid eye movement; SB – Single blinded; SE – Sleep efficiency; SOL – Sleep onset latency; TST – Total sleep time; WASO – Wake after sleep onset.

**Table 2:** The study characteristics and outcomes for the investigations between dietary supplements and sleep.

Author (Year)	Design; Location	Duration; Age; Participants (m/f/t)	Intervention/Control	Sleep Measurement	Outcomes/Results	Notes
Aspy (2018) [31]	RC, DB; Australia	5 dy; 18–40 y; 100 (31/68/1)	B6 (pyridoxine hydrochloride) only (240 mg) OR B complex (range of doses for each B vitamin)/placebo	Subjective (Sleep diary)	Significantly lower sleep quality for B complex compared with B6 only and placebo. Significantly higher tiredness upon waking for B complex compared with B6 only. No significant differences found for vitamin B6.	
Gholipour Baradari (2017) [32]	RC, DB; Iran	1 m; 31.20 ± 5.42 y; 53 (4/49/0)	Zn gelatin capsule/placebo	Subjective (PSQI)	Significant improvement of global sleep score and subjective sleep quality for intervention compared with placebo. Significant improvements within-group for intervention in global sleep score, subjective sleep quality and SOL. Increased zinc serum levels associated with decreased odds of poor sleep.	Intensive care unit nurses, poor sleepers
Romain (2017) [33]	RC, DB, P; Spain	16 wk; 30–50 y; 33 (17/16/0)	HolisFiit supplement (blend of polyphenol compounds; 500 mg)/placebo	Subjective (Athens Insomnia Scale)	Significantly reduced awakening during the night for intervention. Significantly increased total sleep duration, sleep quality, wellbeing during the day and functioning capacity during the day for intervention. Significant improvement in total score for both groups.	Overweight to slightly obese participants (BMI = 25–35 kg/m^2^)
Sarris (2012) [34]	RC, DB, P; Australia	16 wk; 20–50 y; 114 (59/55/0)	Swisse Men’s Ultivite F1W multivitamin or Swisse Women’s Ultivite F1 W multivitamin/placebo	Subjective ratings of sleep-quality	Non-significant increase in SQ Reports of impaired sleep similar between groups	
Wouters-Wesseling (2003) [35]	RC, DB, P; The Netherlands	6 mo; ≥ 65 y; 68 (39/29/0)	Liquid nutrition supplement/placebo	Subjective (Nottingham Health Profile)	Improved sleep quality	
Wightman (2015) [36]	RC, DB, P; UK	28 dy; 18–25 y; 15 (5/10/0)	Pure trans-resveratrol capsule (500 mg; TransmaxTM by BiotiviaTM)/placebo	Subjective (PSQI)	No significant findings for global sleep quality score or individual sleep domains.	
McCarthy (2012) [37]	Single arm trial; US	28 dy; 27.2 ± 1.7 y; 18 (9/9)	Capsule containing Chlorophytum borivilianum and Velvet bean	Subjective (PSQI)	Significant improvements in global sleep quality score and all sleep domains.	
Park (2017) [38]	RC, DB, P, CO; Japan	2 × 5 dy; 25.7 y (mean); 9 (4/5/0)	100ml can containing 600 mg of chlorogenic acid (CGA)/placebo	PSG	Significantly lower SOL for intervention. No significant difference in length of N1 or N2, N3, REM and wakefulness after sleep onset between groups. Significantly higher delta power during first hour of sleep for CGA compared with placebo.	
Yamatsu (2015) [39]	SB, P; Japan	2 × 1 wk; 36.8 ± 8.9; 16 (7/9/0)	Capsules containing GABA OR AVLE OR combination of GABA and AVLE/placebo	PSG	Decrease in SOL for GABA Non-significant decreases in SOL for AVLE and GABA+AVLE. Non-significant decrease in NREM sleep latency for all intervention groups Significant increase in NREM time for AVLE. Non-significant decrease in REM time for all intervention groups.	Poor sleepers
				Subjective (PSQI)	Improved subjective SQ for AVLE	
Kuratsune (2010) [40]	RC, DB, P, CO; Japan	2 × 2 wk; 25–59 yo; 21 (21/0)	Crocetin and dextrin capsule/placebo	Actigraphy	Significantly less wake episodes for intervention compared with placebo No significant difference in SOL or SE.	
				Subjective (St Mary’s Hospital Sleep Questionnaire)	No significant difference in subjective measures.	
Um (2017) [41]	RC, DB, P; South Korea	7 dy; 20 (13/7/0)	Phlorotannin supplement/placebo	PSG	Significant decrease in WASO, difference for TST No significant difference for SOL, SE, TST, REM latency, REM, N1-N3, apnea-hypopnea index or total arousal index between groups.	Participants with self-reported sleep disturbances
				Subjective (PSQI; ESS; Stanford Sleepiness Scale)	Significant decrease in global sleep qual score and daytime functioning for intervention, no difference between groups.Increase in sleep duration.	
Umigai (2018) [42]	RC, DB, CO, P; Japan	2 × 14 dy; 35–60 y; 24 (14/10/0)	Gardenia Yellow capsule with 7.5mg of crocetin/placebo	PSG	Significantly increased delta power for crocetin compared with placebo. No significant differences for SOL, SE, TST, WASO or REM.	Participants with mild sleep complaints All female participants postmenopausal
				Subjective (OSA-MA)	Significant improvements for sleepiness upon rising and feeling refreshed No differences found for sleep duration	
Saito (2017) [43]	RC, DB, P; Japan	12 wk; 20–84 y; 94 (45/74/0)	Placebo (scallop) supplemented with zinc and astaxanthin supplements/placebo	Actigraphy	Significant improvement for SOL compared with placebo. No significant difference in TST, sleep efficiency or body positional changes.	
				Subjective (PSQI; sleep diary)	Significant improvement for global sleep score but not significantly different from placebo.	

CO – Crossover; DB – Double blind; M/F – Male/Female; ESS – Epworth Sleepiness Scale; OSA-MA - Oguri–Shirakawa–Azumi Sleep Inventory, Middle-age and Aged version; P – Placebo; PSG – Polysomnography; PSQI – Pittsburgh Sleep Quality Index; RC – Randomised controlled; REM – Rapid eye movement; SB – Single blinded; TST – Total sleep time; WASO – Wake after sleep onset.

**Table 3:** Study characteristics and outcomes for investigations between food items and sleep.

Author (Year)	Design; Location	Duration; Age; Participants (M/F/T)	Intervention/C	Sleep Measurement	Outcomes/Results	Notes
Garrido (2013) [20]	RC, P, SB, CO	2 × 5 dy; 20–85 y; 30 (15/15/0)	Combination of four cultivars of Jerte Valley cherries, plus 7.5 g maltodextrin and 1.5 g ascorbic acid, diluted	Actigraphy	Significant increase in actual sleep time and immobility for young, middle-aged and elderly groups for intervention compared to baseline. Significant increase in sleep efficiency for elderly groups for intervention with baseline. Significant decrease in number of awakenings and nocturnal activity for young, middle-aged and elderly groups for intervention compared to baseline. Significant decrease in SOL in middle-aged and elderly groups for intervention compared with baseline.	Young, middle-aged and elderly participants
Saito (2017) [43]	RC, DB, P; Japan	12 wk; 20–84 y; 94 (45/74/0)	Zinc-rich food (oysters) OR Zinc- and astaxanthin-rich food (oysters and krill)/placebo	Actigraphy	No significant difference in TST. Significant increase in SE for oysters compared with placebo. Significant improvement in SOL for oysters compared with placebo.	
				Subjective (PSQI)	Significant improvement for sleep scores for all groups, not significantly different from placebo.	
Garrido (2010) [45]	CO; Spain	7 × 3 dy; 35–85 y; 12	Seven cultivars of Jerte Valley cherries ^1^	Actigraphy	Significant increase in actual sleep time for six cultivars in middle-aged group. Significant increase in SE for Van cherries in middle-aged group. Significant decrease in number of awakenings for Pico Limón cherries in middle-aged group and Pico Colorado cherries in elderly group. Significant decrease in total nocturnal activity for Bourlat, Pico Limón, Pico Negro and Pico Colarado cherries for elderly group, and Bourlat cherries for middle-aged group. Significant decrease in SOL for Navalinda cherries in both groups and for Pico Negro cherries in elderly group. Significant increase in assumed sleep for Bourlat, Navalinda and Pico Negro cherries for elderly group, and for Van cherries in middle-aged group. Significant increase in immobility for Ambrunés, Pico Negro and Pico Colorado cherries for elderly group.	Middle-aged and elderly participants
Howatson (2011) [46]	RC, DB, P, CO; UK	7 dy; 18–40 y; 20 (10/10/0)	Tart Montmorency cherry juice (Prunus cerasus) concentrate/placebo	Actigraphy	Significantly greater time in bed, TST and SE total for intervention compared with placebo and baseline. Non-significant decrease in SOL and fragmentation index and non-significant increase in SE for intervention.	
				Subjective (Sleep diaries)	Significantly less napping time for intervention Non-significant increase in SET and TST and non-significant decrease in SOL and WASO for intervention.	

^1^ Cherries cultivated in Jerte Valley, Spain, which contain varied levels of serotonin and melatonin. CO – Crossover; DB – Double blind; M/F/T – Male/Female/Trans; P – Placebo; RC – Randomised controlled; PSQI – Pittsburgh Sleep Quality Index; SB – Single Blind SE – Sleep efficiency; SOL – Sleep onset latency; TST – Total sleep time; WASO – Wake after sleep onset.

**Table 4:** The study characteristics and outcomes for the investigations between macronutrients and sleep.

Author (Year)	Design; Location	Duration; Age; Participants (M/F)	Intervention/Control	Sleep Measurements	Outcomes/Results	Notes
Driver (1999) [47]	RC; South Africa	4 dy; 20–24 y; 7 (7/0)	High energy meal (fat 37%, protein 21%, CHO 42%) OR no meal/control	PSG	No significant difference for TST, SOL, TRT or ROL, or spent in each sleep stage.	
				Subjective (Questionnaire)	No significant difference for subjective sleep quality.	
Afaghi (2007) [48]	RC, CO; Australia	3 × 1 dy; 18–35 y; 12 (12/0)	High GI meals consumed either 4h or 1h before bedtime/control	PSG	Significant shortening of SOL for high GI meal consumed 4 h before bedtime compared with high GI meal consumed 1 hour before bedtime and low GI meal. No significant differences for other sleep parameters between meals or timing of meals.	
Afaghi (2008) [49]	CO; Australia	5 dy; 18–35 y; 14 (14/0)	Very low CHO, high fat, high protein diet (acute and ketosis phases)/control	PSG	Arousal index significantly increased for NREM N1 and N2 during very low CHO phases. No significant difference for N 3 and N4 or REM. Significant increase in total sum of NREM N1-4, N3 and N4 and proportion to TST in very low CHO phases compared with control (due to increases in stage 4). Significant reduction in proportion of REM sleep to TST for very low CHO phases compared with control.	
Lindseth (2011) [50]	RC, DB, CO; US	4 × 4 dy; 19–22 y; 44	High protein diet OR high fat diet OR high CHO diet/control	Actigraphy	Significantly less wake episodes for high protein diet Significantly reduced SOL for high CHO diet No significant differences for high fat diet.	
Yajima (2014) [51]	CO; Japan	2 dy; 24.6 ± 0.7 y; 10 (10/0)	High CHO meal OR high fat meal	PSG	No significant differences when sleep stages were averaged. Significant increase in N3 in first sleep stage for high CHO meal compared with high fat meal.	
Lindseth (2016) [52]	RC, CO; US	4 × 4 dy; 20.9 ± 1.9; 36	High protein OR high fat OR high carbohydrate meals/control	Actigraphy	Significantly shorter wake times after consuming high CHO diet.	
				Subjective (PSQI)	Significantly lower global sleep quality score for high fat diet	
St Onge (2016) [53]	RC, CO; US	5 dy; 30–45 yo; 26 (13/13)	Ad libitum/control	PSG	No significant difference between conditions for TST, absolute time in N1 and N2, and REM sleep. Significant increase of SOL for ad libitum compared with control. Significant reduction in N1 sleep and significant increase in N3 for fibre intake. Significant reduction in N3 for saturated fat. Significant increase in arousals for sugar and other non-sugar/non-fibre CHO.	
Zhou (2016) [54]	RC; CO; US	3 × 4 wk; 14 (3/11)	Energy restricted diets with protein source either beef/pork (BP) or soy/legumes (SL) and either 10% (control), 20% or 30% of intake as protein	Subjective(PSQI)	Improved global sleep quality score for 20% protein intake. No difference in individual sleep domains. No difference in sleep quality between protein source.	Overweight or obese participants (BMI range 27 to 37.9)
Zhou (2016) [54]	RC; DB, P; US	16 wk; 44 (12/32)	High protein energy-restricted diet/control	Subjective (PSQI)	Improvement in global sleep quality score for intervention at week 12 and 16. Greater use of sleep medication for control. No other difference in individual sleep domains.	Overweight or obese participants (BMI range 25 to 38)
Castro (2018) [55]	SA - Spain	4 mo; 18–58 y; 20 (8/12)	High protein diet	Subjective (PSQI; ESS)	Significant improvement in sleepiness mid-intervention (reduced ketosis) No significant difference in sleep quality or sleep duration.	Obese participants (BMI ≥ 30)

BMI – Body mass index; CHO – Carbohydrate; CO – Crossover; DB – Double blind; ESS – Epworth Sleepiness Scale; M/F – Male/Female; NREM – Non-rapid eye movement P – Placebo; PSG – Polysomnography; PSQI – Pittsburgh Sleep Quality Index; RC – Randomised controlled; REM – Rapid eye movement; SOL – Sleep onset latency; TST – Total sleep time.
